# Parameter adaptive terminal sliding mode control for Full-Bridge DC-DC converter

**DOI:** 10.1371/journal.pone.0247228

**Published:** 2021-02-25

**Authors:** Kai Zhou, Chengxiang Yuan, Dongyang Sun, Ningzhi Jin, Xiaogang Wu

**Affiliations:** Engineering Research Center of Automotive Electronics Drive Control and System Integration, Ministry of Education, Harbin University of Science and Technology, Harbin, Heilongjiang, China; National Huaqiao University, CHINA

## Abstract

The poor dynamic performance problem of a Full-Bridge converter under a traditional control strategy is investigated in this study. A new parameter adaptive terminal sliding mode control policy is developed for a Full-Bridge DC-DC converter, by combining the integral part with the power function and differential function in the design of the sliding surface. In theory, the steady-state error of the system can approach zero within a short time. To manage the un-ideal situation after using a fixed value of power γ, an improved γ adaptive algorithm is proposed. The system output is tracked and γ is adjusted in real time. The effect of the system can be guaranteed always in an optimal state. Finally, simulation results are provided to verify the performance of the proposed design method under different conditions.

## 1 Introduction

Driven by the continuous development of new energy vehicles and battery energy storage technology, the Full-Bridge converter has been widely applied in various fields owing to its high power density, high voltage conversion ratio, and low switching loss. Examples include the application of electric vehicle on-board charging systems, DC microgrid energy storage units, and aircraft power supplies [1–3]. Full-Bridge converters are becoming increasingly significant in the application of power electronic circuits. However, traditional Full-Bridge converter circuits present some inherent defects, such as the loss of the duty cycle and the voltage spike on the rectifier output of the secondary side during the operation phase of the circuit. Many scholars have proposed improvement schemes to overcome the above-mentioned shortcomings. For example, diodes can be connected in series in an inverter circuit on its primary side [4], and a midpoint clamp circuit can be introduced in the subsequent stage of the secondary-side rectifier circuit [5]. Although many established solutions can be used to solve this problem, an optimal solution does not exist for solving the poor dynamic performance of the Full-Bridge converter under traditional control strategies, thereby significantly limiting the application of the Full-Bridge converter. Therefore, this study aims to provide a control strategy with strong dynamic performance and superior regulation performance.

Owing to the emergence of classic control theory in the early 1940s, the application of frequency domain and root locus methods has transformed engineering object analysis from the time domain to the frequency domain; additionally, a system closed-loop control based on a PID controller was enabled. Meanwhile, the emergence of modern control theory at the end of the 20th century has contributed to the realization and subsequently application of neural networks [6]. Furthermore, the new control theory [7, 8] has been applied to intelligent control in various engineering applications, such as network communication [9–11], actuator fault analysis [12]. Hence, if the traditional PID control is emphasized instead of innovations consistent with theoretical developments during the analysis of the power electronic circuit topology, then improvements to the overall control effect of the system would be hindered significantly. It is undeniable that the PID controller offer many advantages, such as simple design, convenient application, and easy hardware implementation. However the PID controller presents many problems as well, such as slow startup speed and long load adjustment time. Consequently, a controller with a simple application and superior dynamic performance must be designed.

Currently, studied regarding new control strategies for circuit topologies are primarily focused on the Buck circuit, whereas the Full-Bridge circuit is rarely reported. Control-restricted sliding mode controller design [13] and fuzzy PI controller design [14] proposed for the Buck circuit has achieved favorable control effects. Furthermore, sliding mode control theory is the most typically used theory in situations involving high system recovery requirements under rapid disturbance because of the simple design and strong robustness of the sliding surface. However, the control strategy designed for the Buck circuit cannot be directly applied to the Full-Bridge converter, which requires the transformation of an equivalent control amount. This poses a significant problem that restricts the development of Full-Bridge converter control strategies.

Scholars have provided their own solutions and achieved good results. For example, in [15], the full-order sliding mode control strategy can be applied to the Full-Bridge converter and, improved the robustness and regulation performance of the system. Bo H [16] proposed a backstepping sliding mode control method suitable for Full-Bridge inverters, thereby reducing the dependence of the controller on the system and improving the universality of the controller. In [17], a double integral indirect sliding mode control strategy was proposed to eliminate the output residual caused by indirect sliding mode control by adopting a double integral, which improved the dynamic quality of the converter. However, the control methods above depend significantly on parameters. The setting of different parameters significantly affects the control effect of the system. The system does not exhibit an optimal control effect when it is disturbed. Hence the applicability of the algorithm must be improved under different operating conditions. A terminal sliding-mode control strategy using parameter adaptation is proposed herein. The power coefficient of the sliding mode surface can be adjusted in real time based on the output of the system status. The self-adjustment of the γ parameter was realized through the designed γ parameter adaptive algorithm, thereby eliminating the dependence of parameters on the system operating conditions and ensuring the optimal system control effect under all operating conditions.

Based on the discussion and analysis above, a terminal sliding mode controller with a simple application, strong dynamic recovery performance, and parameter adaptation for Full-Bridge converters was designed in this study. The main contributions of this study are as follows:

1. A mathematical model is established for a Full-Bridge circuit using state-space-cycle averaging based on the circuit characteristics of the Full-Bridge converter, thereby providing a foundation for the theoretical analysis of subsequent controller designs.
2. A sliding mode with a strong anti-disturbance and adjustable control parameters is designed. An equivalent control variable expression suitable for Full-Bridge converters is derived. Stability analysis is performed on the designed sliding mode surface. The steady-state time of the system is calculated theoretically.
3. A γ parameter adaptive algorithm is proposed, and the λ factor is introduced. The optimal γ algorithm expression is determined, whereas the system control effects under different conditions are compared.

The remained of this paper is organized as follows: Section 2 introduces the mathematical model of the Full-Bridge converter. Section 3 presents the design of the terminal sliding surface for the Full-Bridge converter as well as a proof of its existence and the system steady-state time. Section 4 presents the improved γ adaptive algorithm, a comparison of different γ values based on an adaptive algorithm, as well as the simulation results. Section 5 presents the conclusions and future studies.

## 2 Modeling of Full-Bridge converter

**Fig 1:**
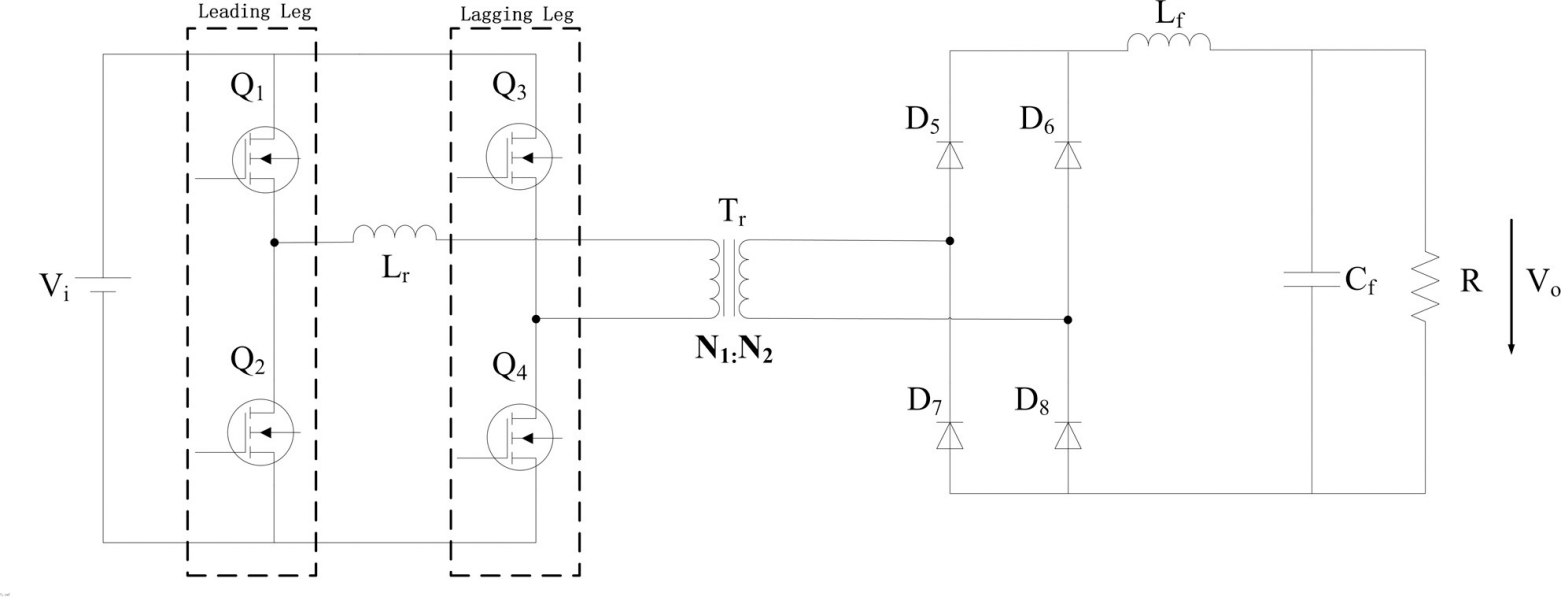
Basic circuit topology of Full-Bridge converter.

The topology of the Full-Bridge converter is shown in (Fig 1). V_i_ is the input voltage. L_r_ is the primary resonant inductor, L_f_ and C_f_ are the filter inductance and filter capacitance respectively, R is the load resistance, and V_o_ is the load output voltage. MOSFETs Q_1_ and Q_2_ are leading legs, and Q_3_ and Q_4_ are lagging legs. Diodes D_5_−D_8_ constitute the uncontrolled rectifier in the circuit secondary side. The primary and secondary turns of the isolation transformer T_*r*_ are *N*_1_ and *N*_2_, respectively, and can be expressed as $K=\frac{N_{1}}{N_{2}}$.

**Fig 2:**
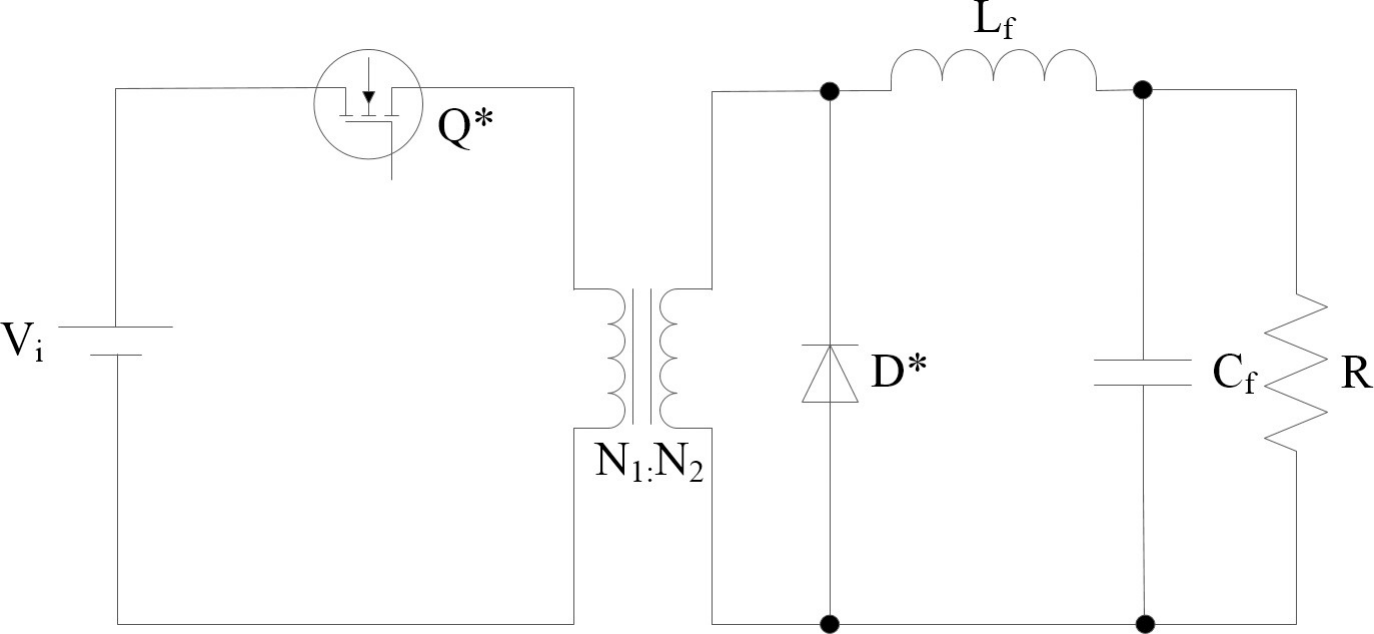
Buck circuit equivalent model of Full-Bridge converter.

Because the Full-Bridge converter can be regarded as a circuit derived from the Buck circuit, the mathematical model of the Full-Bridge converter can be based on the modeling method of the Buck circuit; the equivalent Buck circuit model is shown in (Fig 2).

where Q*and D* represent the equivalent MOSFET and diode of the Buck converter, respectively. When Q* = 1, the switch is ON, and when Q* = 0, it is OFF. By performing period averaging in the state space of the Buck circuit, the equivalent model of the Full-Bridge converter can be obtained as follows [18]:

$$Q*=1:\frac{d_{i_{L}}}{d_{t}}=\frac{V_{i}}{KL_{f}}-\frac{V_{o}}{L_{f}},\;\frac{d_{V_{o}}}{d_{t}}=\frac{i_{L}}{C_{f}}-\frac{V_{o}}{RC_{f}}$$ (1)

$$Q*=0:\frac{d_{i_{L}}}{dt}=-\frac{V_{o}}{L_{f}},\;\frac{d_{V_{o}}}{d_{t}}=\frac{i_{L}}{C_{f}}-\frac{V_{o}}{RC_{f}}$$ (2)

where *i*_*L*_ is the inductor current. The equivalent switch value u is introduced to describe the Q* state, i.e. 1 for the ON state and 0 for the OFF state.

$$\frac{d_{i_{L}}}{d_{t}}=\frac{V_{i}}{KL_{f}}u-\frac{V_{o}}{L_{f}}$$ (3)

$$\frac{d_{V_{o}}}{d_{t}}=\frac{i_{L}}{C_{f}}-\frac{V_{o}}{RC_{f}}$$ (4)

The output voltage error *x*_1_ is defined as follows:

$$x_{1}=V_{o}-V_{ref}$$ (5)

where *V*_*ref*_ is the output voltage reference value. By taking the time derivative of Eq (5), *x*_2_ is defined as the rate of change of voltage error, which can be expressed as

$$x_{2}=\dot{x_{1}}=\dot{V_{o}}-\dot{V_{ref}\approx }\dot{V_{o}}$$ (6)

Subsequently, the equivalent state-space model of the Full-Bridge converter can be expressed as

$$\left[\begin{array}{c} \dot{x_{1}}\\ \dot{x_{2}} \end{array}\right]=\left[\begin{array}{cc} 0 & 1\\ -\frac{1}{L_{f}C_{f}} & -\frac{1}{RC_{f}} \end{array}\right]\left[\begin{array}{c} x_{1}\\ x_{2} \end{array}\right]+\left[\begin{array}{c} 0\\ \frac{uV_{i}-KV_{ref}}{KL_{f}C_{f}} \end{array}\right]$$ (7)

## 3 Terminal sliding mode control for Full-Bridge converter

The traditional sliding mode surface is typically selected as a linear plane. However, a linear sliding surface presents some inherent shortcomings. For example, regardless of the adjustment of the sliding mode parameters, the state tracking error of the system will not converge to zero in a finite time. Hence, a nonlinear function was introduced into the design of the sliding surface [19, 20]. Based on the integral characteristics of the nonlinear function, the system state tracking error gradually approaches 0. The proposed function S can be defined as follows:

$$S=\dot{x_{1}^{\gamma }}+k_{a}x_{1}^{\gamma }+k_{b}\int_{0}^{t}x_{1}^{\gamma }d\tau$$ (8)

$$k_{a}<0,k_{b}<0,0<\gamma <1$$ (9)

where *k*_*a*_ and *k*_*b*_ represent the sliding mode coefficients. The specific value ranges of *k*_*a*_ and *k*_*b*_ and the effect of their parameter changes on the system control effect will be provided in Section 3.1.

### 3.1 Proof of system stability

According to the Lyapunov stability criterion, to guarantee the stability of a system, in terms of the sliding mode control, the state trajectory of the system may be stable on the sliding surface and shift to the origin after reaching the sliding surface. Additionally, the system status must satisfy the following requirements [21].

$$\underset{S\to 0}{\lim}S\dot{S}<0$$ (10)

Subsequently, the Lyapunov function is expressed as

$$V=\frac{1}{2}S^{2}$$ (11)

The time derivative of Eq (11) can be written as

$$\dot{V}=S\dot{S}$$ (12)

Subsequently the derivative of Eq (8) is calculated.

When u = 1 and S<0, we can obtain the following based on Eq (7):

$$\dot{x_{1}}=\frac{1}{C_{f}}(i_{L}-\frac{V_{o}}{R})$$ (13)

$$\ddot{x_{1}}=\frac{\left(V_{i}-KV_{o}\right)}{KL_{f}C_{f}}-\frac{1}{RC_{f}^{2}}(i_{L}-\frac{V_{o}}{R})$$ (14)

By deriving the Eq (8) and introducing it into Eqs (13) and (14), the following results are obtained:

$$\begin{array}{l} \dot{S}=\frac{\gamma }{C_{f}^{\gamma -1}}{\left(i_{L}-\frac{V_{o}}{R}\right)}^{\gamma -1}\left[\frac{\left(V_{i}-KV_{o}\right)}{KL_{f}C_{f}}-\frac{1}{RC_{f}^{2}}(i_{L}-\frac{V_{o}}{R})\right]+k_{a}\gamma x_{1}^{\gamma -1}\frac{1}{C_{f}}\left(i_{L}-\frac{V_{o}}{R}\right)+k_{b}x_{1}^{\gamma }\\ \;=\frac{(V_{i}-KV_{o})\gamma }{KL_{f}C_{f}^{\gamma }}{\left(i_{L}-\frac{V_{o}}{R}\right)}^{\gamma -1}-\frac{\gamma }{RC_{f}^{\gamma +1}}{\left(i_{L}-\frac{V_{o}}{R}\right)}^{\gamma }+k_{a}\gamma x_{1}^{\gamma -1}\frac{1}{C_{f}}\left(i_{L}-\frac{V_{o}}{R}\right)+k_{b}x_{1}^{\gamma }>0 \end{array}$$ (15)

When u = 0, S>0 and we can obtain the following based on Eq (7):

$$\dot{x_{1}}=\frac{1}{C_{f}}(i_{L}-\frac{V_{o}}{R})$$ (16)

$$\ddot{x_{1}}=-\frac{V_{o}}{L_{f}C_{f}}-\frac{1}{RC_{f}^{2}}(i_{L}-\frac{V_{o}}{R})$$ (17)

Calculating the derivative of Eq (8) and then substituting it into Eqs (16) and (17) yields.

$$\begin{array}{l} \dot{S}=\frac{\gamma }{C_{f}^{\gamma -1}}{\left(i_{L}-\frac{V_{o}}{R}\right)}^{\gamma -1}\left[-\frac{V_{o}}{L_{f}C_{f}}-\frac{1}{RC_{f}^{2}}(i_{L}-\frac{V_{o}}{R})\right]+k_{a}\gamma x_{1}^{\gamma -1}\frac{1}{C_{f}}\left(i_{L}-\frac{V_{o}}{R}\right)+k_{b}x_{1}^{\gamma }\\ \;=-\frac{\gamma V_{o}}{L_{f}{C_{f}}^{\gamma }}{\left(i_{L}-\frac{V_{o}}{R}\right)}^{\gamma -1}-\frac{\gamma }{RC_{f}^{\gamma +1}}{\left(i_{L}-\frac{V_{o}}{R}\right)}^{\gamma }+k_{a}\gamma x_{1}^{\gamma -1}\frac{1}{C_{f}}\left(i_{L}-\frac{V_{o}}{R}\right)+k_{b}x_{1}^{\gamma }<0 \end{array}$$ (18)

By setting γ≈1 and i_L_≈0, the conditions that limit the existence region of the design parameters can be obtained as follows:

$$\frac{1}{RC_{f}}-\frac{R}{L_{f}}<k_{a}<0$$ (19)

$$\frac{{\left(RC_{f}\right)}^{2}-1+RC_{f}k_{a}}{{\left(RC_{f}\right)}^{3}}<k_{b}<0$$ (20)

Subsequently, we can obtain the specific value range of *k*_*a*_ and *k*_*b*_. As shown in Eqs (19) and (20), the values of *k*_*a*_ and *k*_*b*_ are affected by the circuit parameters. Different circuits have different values of *k*_*a*_ and *k*_*b*_, where *k*_*a*_ is the coefficient before the deviation term. Increasing *k*_*a*_ can improve the corresponding speed of the system. However, the overshoot of the system may increase if the value is excessively high. Meanwhile, *k*_*b*_ is the coefficient before the integral term. Increasing *k*_*b*_ can shorten the steady-state adjustment time of the system, whereas if *k*_*b*_ is excessively large, integral oversaturation may occur in practical applications. Therefore, the selection of control parameters significantly affects the final control effect of the system.

Using Eqs (15) and (18) and introducing the equivalent control quantity u_eq_, we can obtain

$$\dot{S}=\frac{\gamma V_{i}}{KL_{f}C_{f}^{\gamma }}{\left(i_{L}-\frac{V_{o}}{R}\right)}^{\gamma -1}u_{eq}-\frac{\gamma V_{o}}{L_{f}C_{f}^{\gamma }}{\left(i_{L}-\frac{V_{o}}{R}\right)}^{\gamma -1}-\frac{\gamma }{RC_{f}^{\gamma +1}}{\left(i_{L}-\frac{V_{o}}{R}\right)}^{\gamma }+k_{a}\gamma x_{1}^{\gamma -1}\frac{1}{C_{f}}\left(i_{L}-\frac{V_{o}}{R}\right)+k_{b}x_{1}^{\gamma }$$ (21)

If Eq (21) yields 0, then the equivalent control law u_eq_ can be written as

$$u_{eq}=\frac{K}{V_{i}}\left[V_{o}+\frac{L_{f}}{RC_{f}}(i_{L}-\frac{V_{o}}{R})-k_{a}L_{f}C_{f}^{\gamma -1}{x_{1}^{\gamma -1}(i_{L}-\frac{V_{o}}{R})}^{2-\gamma }-\frac{L_{f}k_{b}}{\gamma }{\left(C_{f}x_{1}\right)}^{\gamma }{\left(i_{L}-\frac{V_{o}}{R}\right)}^{1-\gamma }\right]$$ (22)

Eq (22) shows that if we set γ = 1, the expression of the control law u_eq_ degenerates into the second-order sliding mode control, considering that the actual γ parameter is dynamic; however, because the setting time is typically extremely short, it can be regarded as a constant. Because γ∈(0,1), the time complexity of the proposed control algorithm can be expressed as T(n) = O(x^γ^). However, the time complexity of the algorithm with a fixed γ parameter is expressed as T_2_(n) = O(x). Therefore, if only the time complexity is considered, then the time complexity of the proposed algorithm will be less, whereas the theoretical execution efficiency will be higher.

**Fig 3:**
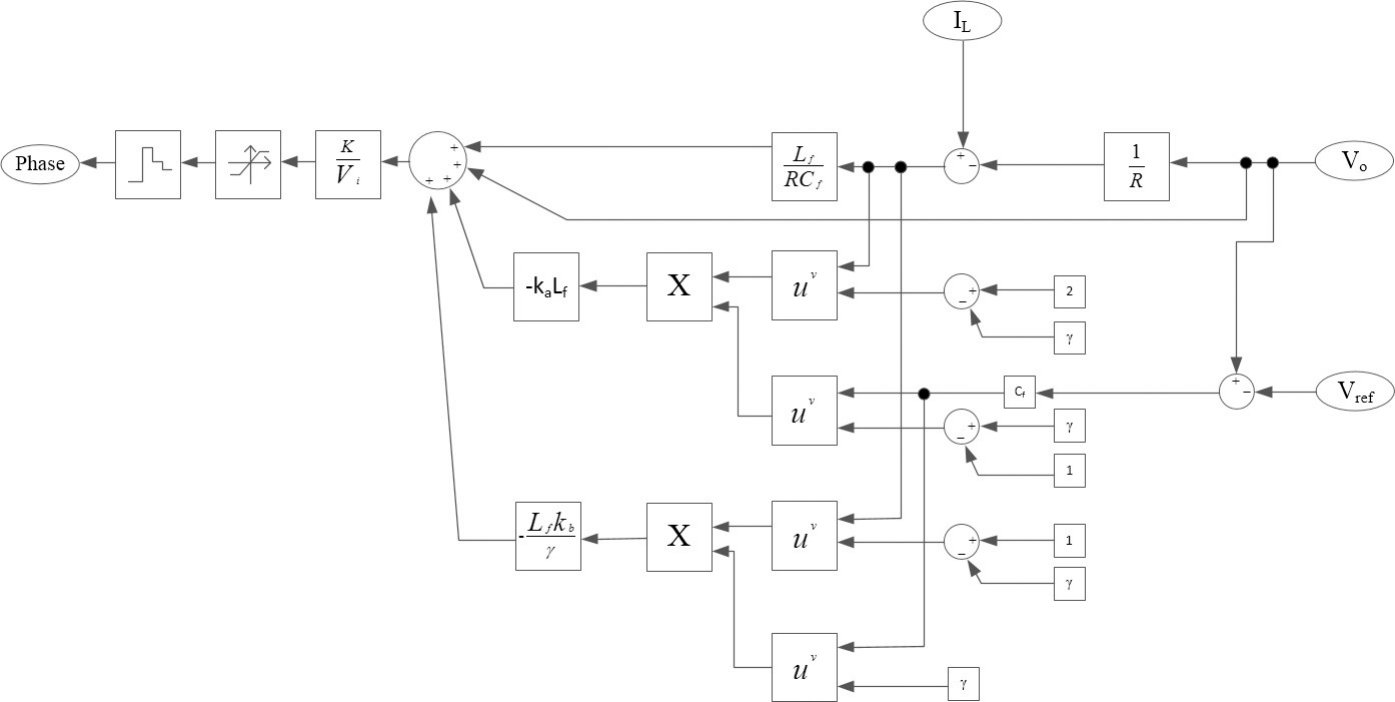
System block diagram of control law u_eq_.

The block diagram of the control law u_eq_ is shown in (Fig 3).

### 3.2 Analysis of system sliding mode arrival time

The sliding mode theory claims that, when a system is stable on a sliding surface, then the equation of the sliding surface satisfies S = 0. Based on Eq (8), we assume that

$$f=\int_{0}^{t}x_{1}^{\gamma }d\tau$$ (23)

When S = 0, Eq (8) can be rewritten as

$$k_{a}x_{1}^{\gamma }=-\dot{x_{1}}\gamma -k_{b}f$$ (24)

By further simplification, we obtain

$$k_{a}\dot{f}=-\frac{\gamma }{C_{f}}i_{c}(t)-k_{b}f$$ (25)

where i_c_(t) represents the resonant capacitance current expansion from Eq (25). Subsequently,

$$k_{a}\frac{df}{dt}=-\frac{\gamma }{C_{f}}i_{C}(t)-k_{b}f$$ (26)

$$\frac{C_{f}k_{a}}{-\gamma i_{C}(t)-C_{f}k_{b}f}df=dt$$ (27)

Taking the integral of both sides of Eq (27) from t∈[0, t_fin_], noting that x_1_(0)≠0, x_1_(t_fin_) = 0, the finite time t_fin_ can be expressed as

$$t_{fin}=|-\frac{k_{a}}{k_{b}}\ln\left(-\gamma i_{C}-\frac{C_{f}k_{b}}{1+\gamma }{x_{1}(0)}^{1+\gamma }\right)|$$ (28)

As shown in Eq (28), t the convergence time t_fin_ depends on the parameters of k_a_,k_b_,γ. Therefore, a reasonable value must be selected to ensure the stability and improve the regulation time of the system.

## 4 Adaptive strategy of γ and simulation results

We used MATLAB/Simulink to verify the proposed theory. The system simulation framework diagram is shown in S1 Fig, and the simulation model of u_eq_ is shown in S2 Fig. The parameters of Full-Bridge converter are provided in Table 1.

**Table 1:** Simulation parameters of Full-Bridge converter.

Description	Parameters	Nominal values
Input voltage	*V*_*i*_	24VDC
Desired output voltage	*V*_*ref*_	14VDC
Inductance	*L*_*f*_	50μH
Capacitance	*C*_*f*_	500μF
Load resistance	R	2.2Ω

As shown in Eq (22), when γ = 1, u_eq_ degenerated into the second-order sliding mode control, i.e.,

$$u_{eq}=\frac{KV_{o}}{V_{i}}+\frac{KL_{f}}{V_{i}}i_{c}\left(\frac{1}{RC_{f}}-k_{a}\right)-\frac{KL_{f}C_{f}k_{b}}{V_{i}}x_{1}$$ (29)

**Fig 4:**
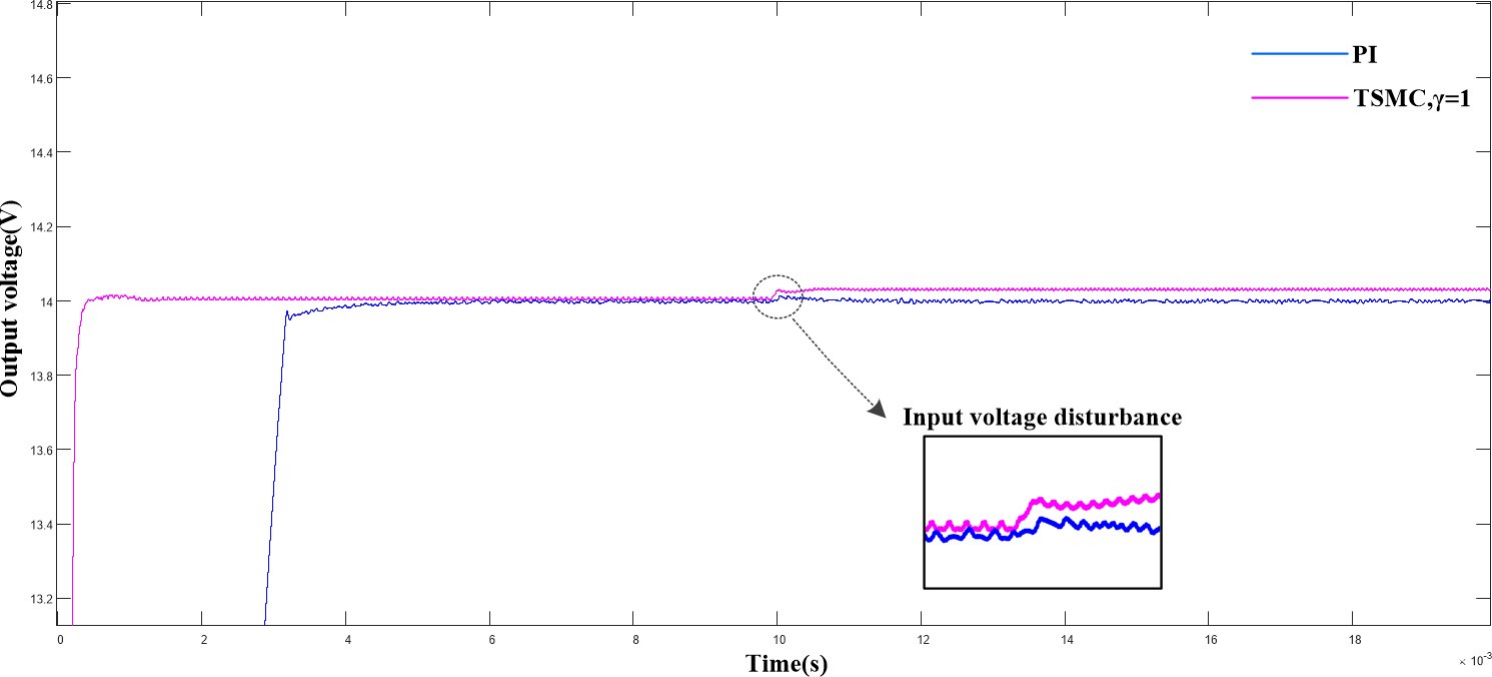
Comparison of output voltage waveform between double closed loop PI control and terminal sliding mode control under input voltage disturbance.

**Fig 5:**
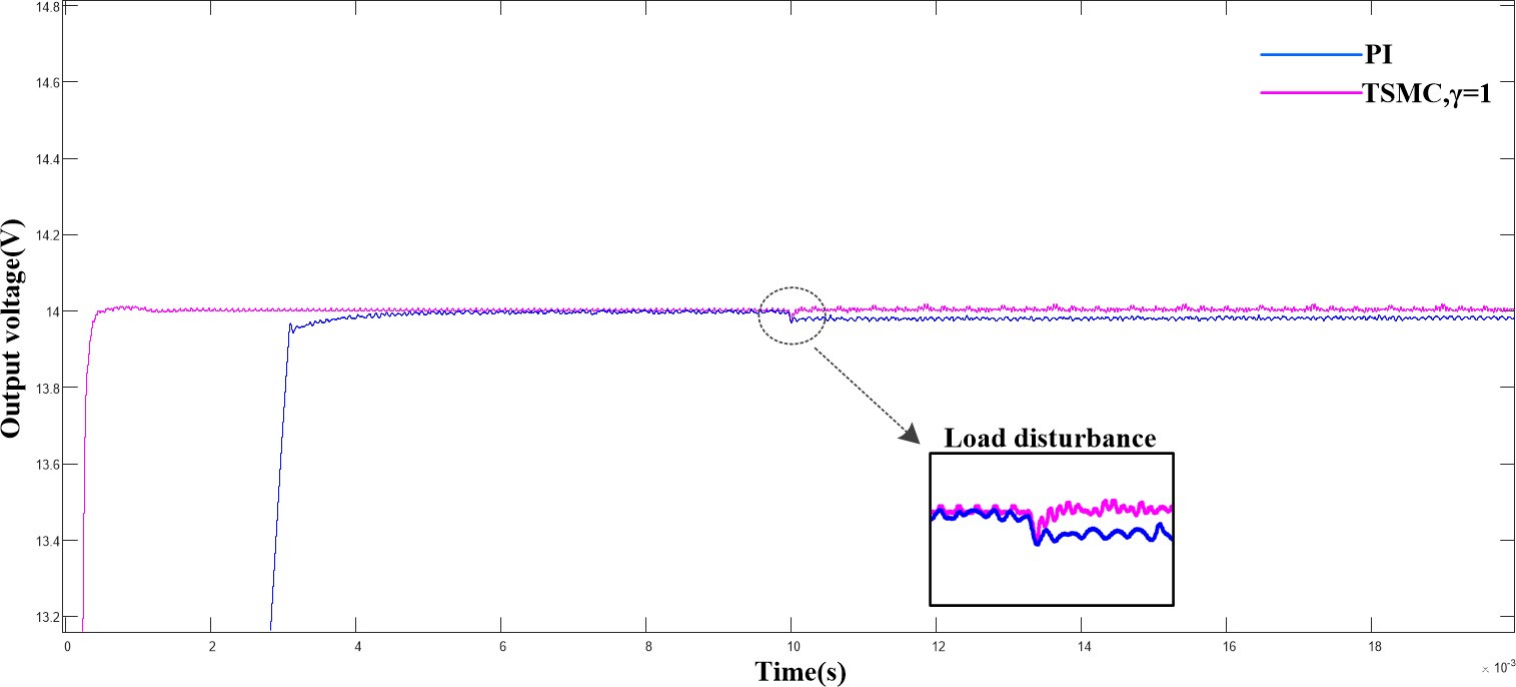
Comparison of output voltage waveform between double closed loop PI control and terminal sliding mode control under load disturbance.

**Fig 6:**
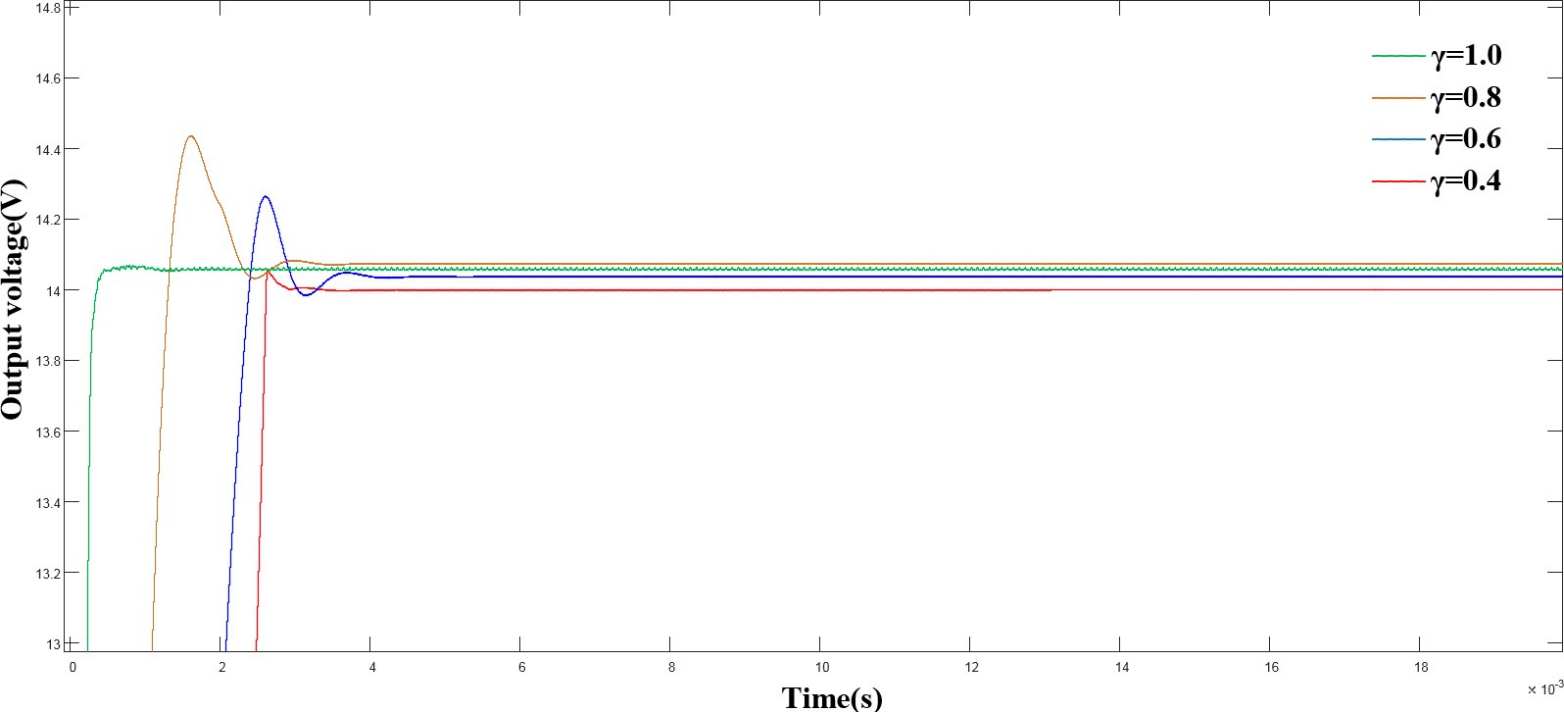
Comparison of output voltage under different γ values.

In this study k_a_ = −4×10^4^ and k_b_ = −3×10^10^ were selected and substituted into Eq (29). Compared with the traditional PI control, the output voltage waveforms were observed based on simulations, as shown in (Fig 4).

(Fig 4) shows a comparison of the output voltage waveform under the input voltage disturbance of the terminal sliding mode control with double closed-loop PI control. As shown, compared with the traditional PI control, the terminal sliding mode control exhibits better rapidity in the start-up stage. It required only 1.3 ms to achieve a steady-state output, and, 4.2 ms for double closed-loop PI control. The input voltage was stepped up from 24 VDC to 26 VDC in 0.01 s. Although neither of the two control models reached the pre-steady-state, the output voltage was still within the allowable voltage error range.

(Fig 5) shows the output voltages of the PI control and terminal sliding mode control when the load changed abruptly at 0.01 s. The output voltage waveform shows, that the output voltage under the terminal sliding mode control strategy after 62 μs of regulation time stabilized, with a steady-state error of 0. However, the system could not reach its initial state under the PI control. Combined with the experimental results shown in (Figs 4 and 5) and Table 2, we verified that the proposed terminal sliding mode control strategy is superior to the traditional PI control strategy in terms of rapidity and robustness. Although γ = 1 was specified, different values of γ imposed different effects on the system performance. (Fig 6) shows a comparison of the output voltage when γ = 0.4, 0.6, 0.8, and 1.0.

**Table 2:** Comparison between PI control and terminal sliding mode control in different disturbance scenarios.

Control mode	Rise time(ms)	Setting time(ms)	Disturbance in regulation time(μs)	Disturbance in voltage drop(mV)	Steady-state error(mV)
PI	4.2	5.1	50 (V)、73(L)	-1(V)、2(L)	1(V)、2(L)
Terminal Sliding Mode Control	1.3	1.5	37(V)、62(L)	-3(V)、1.5(L)	3(V)、0(L)

^a^In Table 2 ‘‘(V)” represents the experimental results under voltage disturbance, and ‘‘(L)” represents the experimental results under load disturbance.

**Fig 7:**
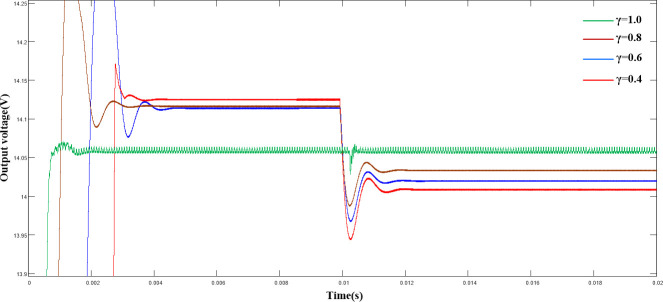
Comparison of output voltage waveforms with different γ values under load disturbance.

(Fig 6) shows a comparison of the output voltage under different γ. As shown, as the γ value increased, the response speed of the system increased and the rise time of the system decreased. Additionally, a significant voltage overshoot was generated, resulting in reduced stability in the system.

(Fig 7) shows a comparison of the output voltage under load disturbance with different γ values. As shown, as γ decreased, the overshoot in the start-up phase of the system decreased correspondingly, but a more significant voltage drop occurred. Combined with the experimental results in (Figs 6 and 7), we discovered that a fixed γ cannot guarantee the optimal control state of the system. Therefore, an improved γ-adaptive algorithm is proposed.

By analyzing the system state, we discovered that when the system approached the steady state, i.e., as |x_1_| approached to 0, the convergence speed of $\left|x_{1}^{\gamma }\right|$ was higher than of |x_1_|; meanwhile, when |x_1_| was greater than 1, the convergence speed of $\left|x_{1}^{\gamma }\right|$ was slower than that of |x_1_|.Our aim is to identify an algorithm where the value of γ can be minimal when |x_1_|<1, such that the system can approach to the steady state more quickly. When |x_1_|>1, the value of γ can be approximately 1, which is similar to the linear approach effect. In Ref. [22], a γ adaptive algorithm was presented, i.e., $\gamma =\frac{1}{\pi }\arctan\left(x_{1}-0.99\right)+0.5$; however, the proposed algorithm, could not guarantee that |x_1_| would yield the fastest approaching speed at x∈[0,1]. Therefore, an improvement to the γ adaptive algorithm using parameter λ is proposed herein.

$$\gamma =\frac{1}{\pi }\arctan\left({\lambda x}_{1}-1\right)+0.5(\lambda >0)$$ (30)

**Fig 8:**
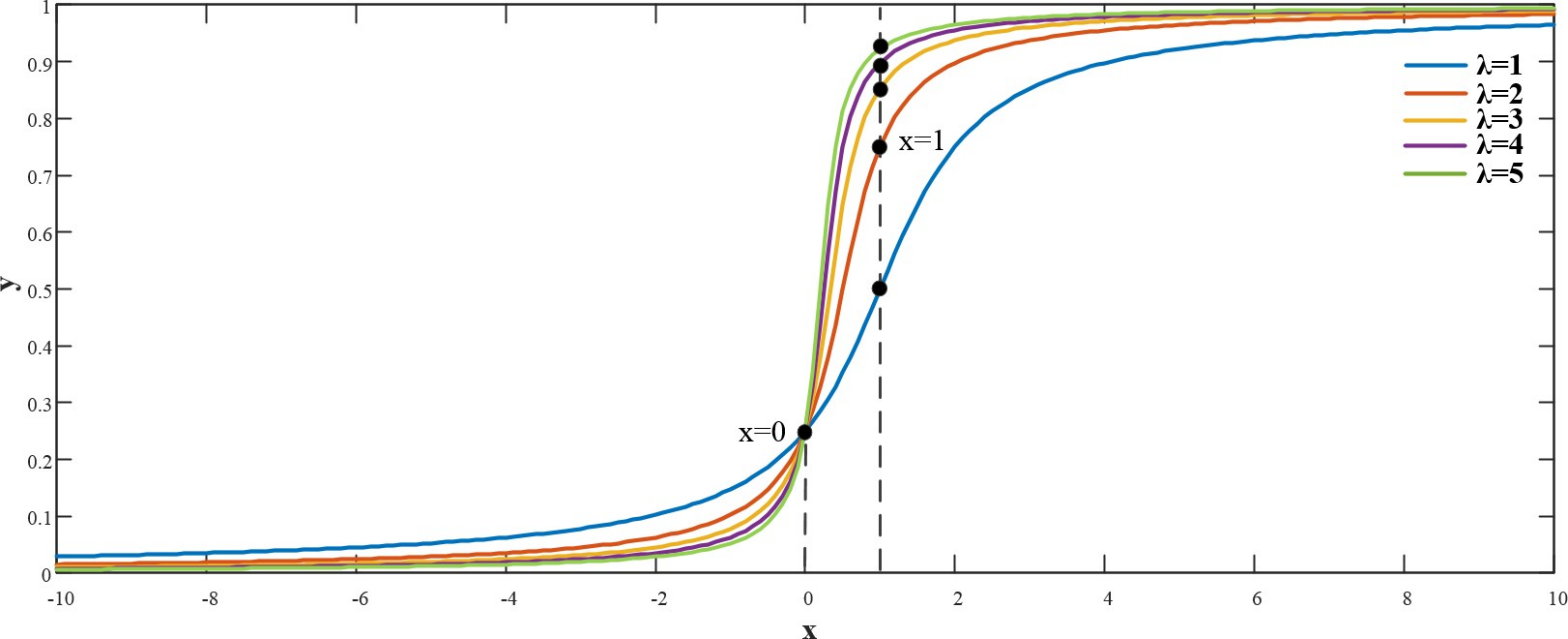
γ function with different λ values.

**Fig 9:**
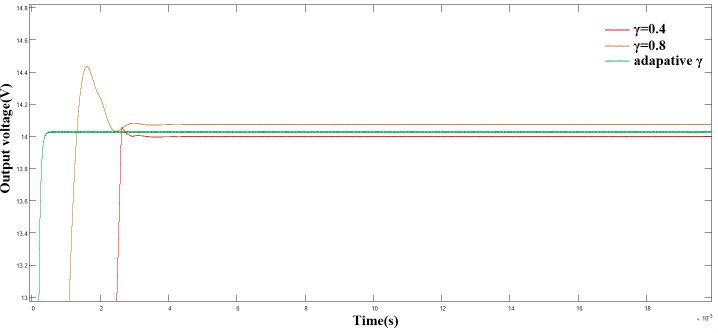
Comparison of output voltage with adaptive algorithm.

**Fig 10:**
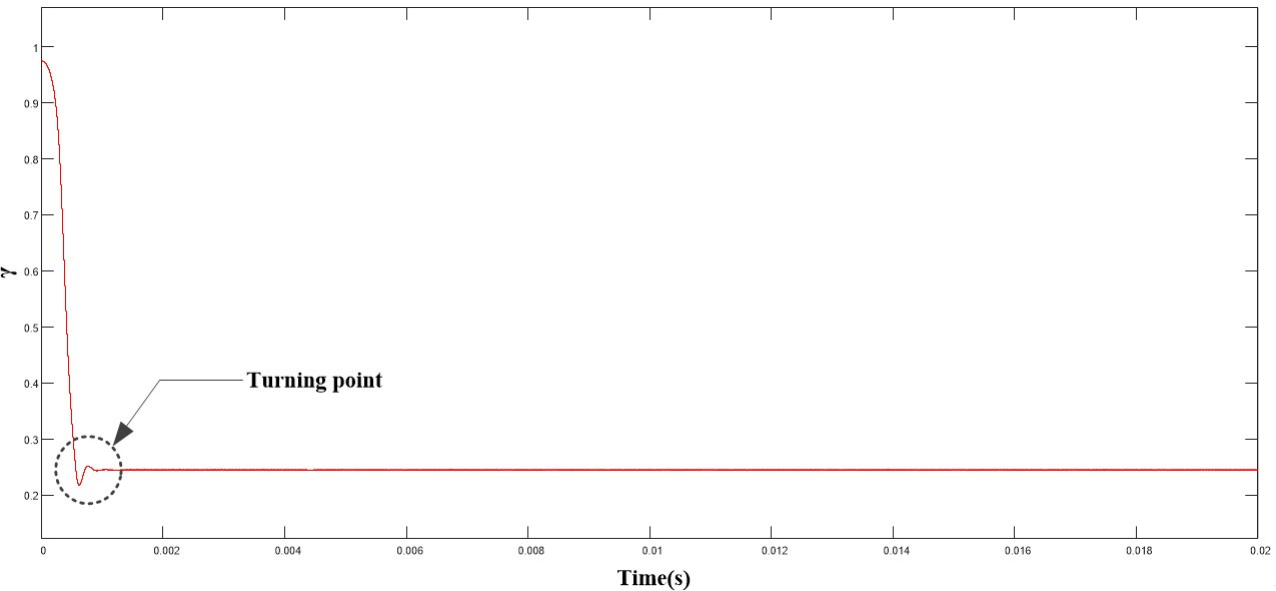
Variation curve of γ parameter.

**Fig 11:**
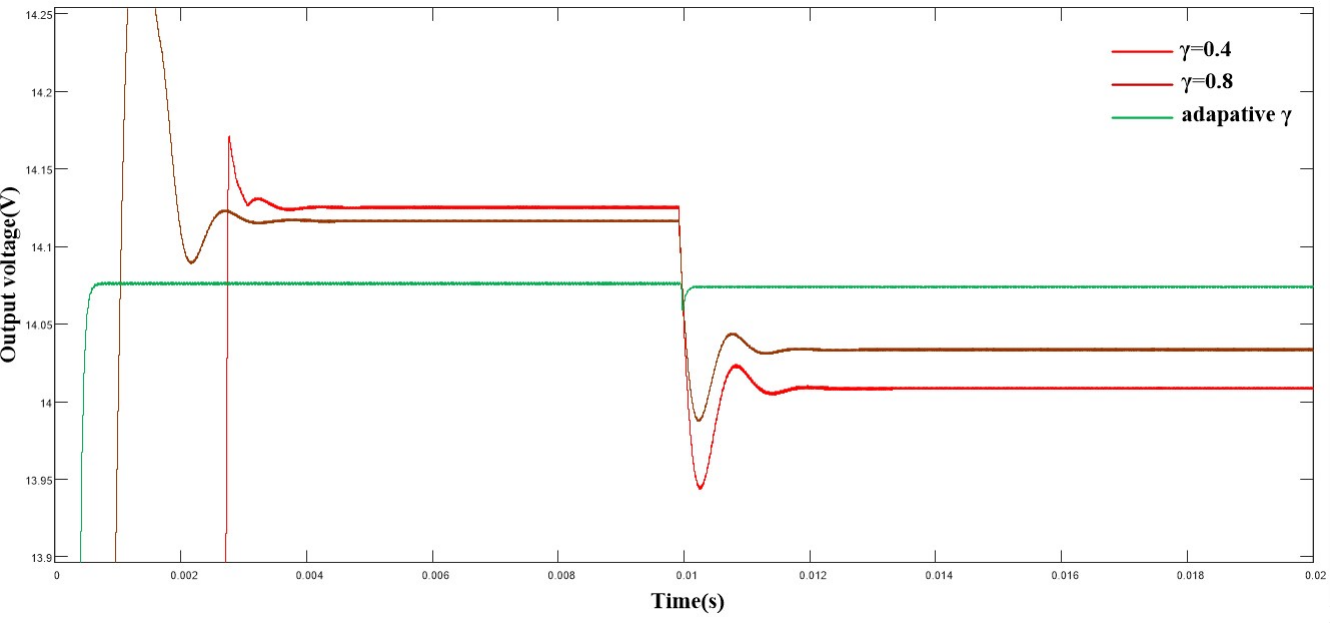
Comparison of output voltage with adaptive algorithm under load disturbance.

**Fig 12:**
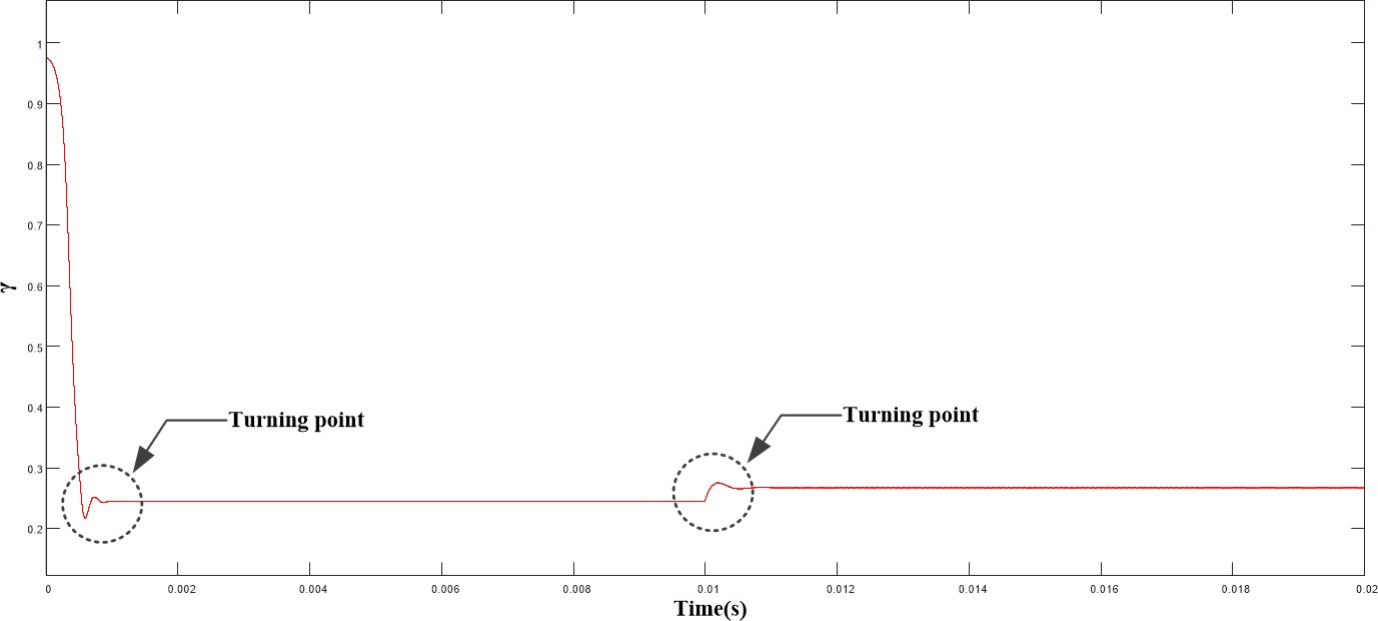
Variation curve of γ under load disturbance.

As shown in (Fig 8), the slope of the function on x∈[0,1] increased rapidly with λ. Furthermore, when | x | > 1, the function approached 1 at a higher speed, consistent with the expected results. However, if λ becomes excessively large, then the stability of the system may be impaired. Therefore, to consider the stability and rapidity of the system, here we set λ = 4.

(Fig 9) shows a comparison of the output voltage with γ = 0.4, and γ = 0.8, where γ uses the adaptive algorithm. As shown, compared with the fixed γ value, the adaptive algorithm can track the system state more effectively and improve the rapidity and stability of the system, while reducing the overshoot of the system.

(Fig 10) shows the variation curve of the γ parameter. As shown, as |x_1_| decreased, γ decreased simultaneously, and stabilized after reaching the turning point, consistent with the theoretical analysis.

(Fig 11) shows the output voltage between a fixed γ and an adaptive value when the load is disturbed in 0.01 s. Compared with the fixed value, the output voltage drop of the system reduced significantly. Compared with the situation where γ = 0.4, the voltage drop with the adaptive algorithm reduced by almost 130 mV, and the regulation time reduced significantly, thereby achieving a better control effect.

(Fig 12) shows the variation curve of γ. As shown, γ parameter reached the steady state rapidly (0.5 ms) after a short adjustment in the start-up stage and then remained stable. Hence, the control effect of the system was ensured.

## 5 Conclusions

To improve the performance of the Full-Bridge converter and improve the anti-interference capability of the system, a parameter adaptive terminal sliding mode control strategy was proposed herein. Simulation results verified that the proposed control strategy yielded better results than the traditional PI control in terms of speed and robustness. Because the fixed γ parameter could not consider all aspects of the system’s fast and dynamic adjustment performance, an improved adaptive algorithm for γ was proposed herein based on previous studies. By introducing λ, the original γ adaptive algorithm can be changed automatically in different |x_1_|. The proposed approach improved the dynamic regulation performance of the system. Simulation results showed that the proposed adaptive terminal sliding mode control strategy can achieve better dynamic performances and exhibited better recovery characteristics when disturbance occurred. In addition to the sliding mode control theory, there are still many excellent theoretical ideals that can be applied to the Full-Bridge circuits. Such as the switching technology and multiple Lyapunov functions method. All of these discussed can be regarded as our future research directions, so as to design a Full-Bridge circuit with better performance and stronger applicability.

## Supporting information

S1 Fig System simulation framework diagram. The overall design framework is given in this figure.
(TIF)

S2 Fig Simulation model of u_eq_. Here is the specific framework of *u*_*eq*_ in MATLAB/Simulink.
(TIF)
