# Supplementary figures and images for: Parameter adaptive terminal sliding mode control for Full-Bridge DC-DC converter
Source: PLoS One. 2021 Feb 25;16(2):e0247228. doi: 10.1371/journal.pone.0247228 (PMC7906401; doi:10.1371/journal.pone.0247228)

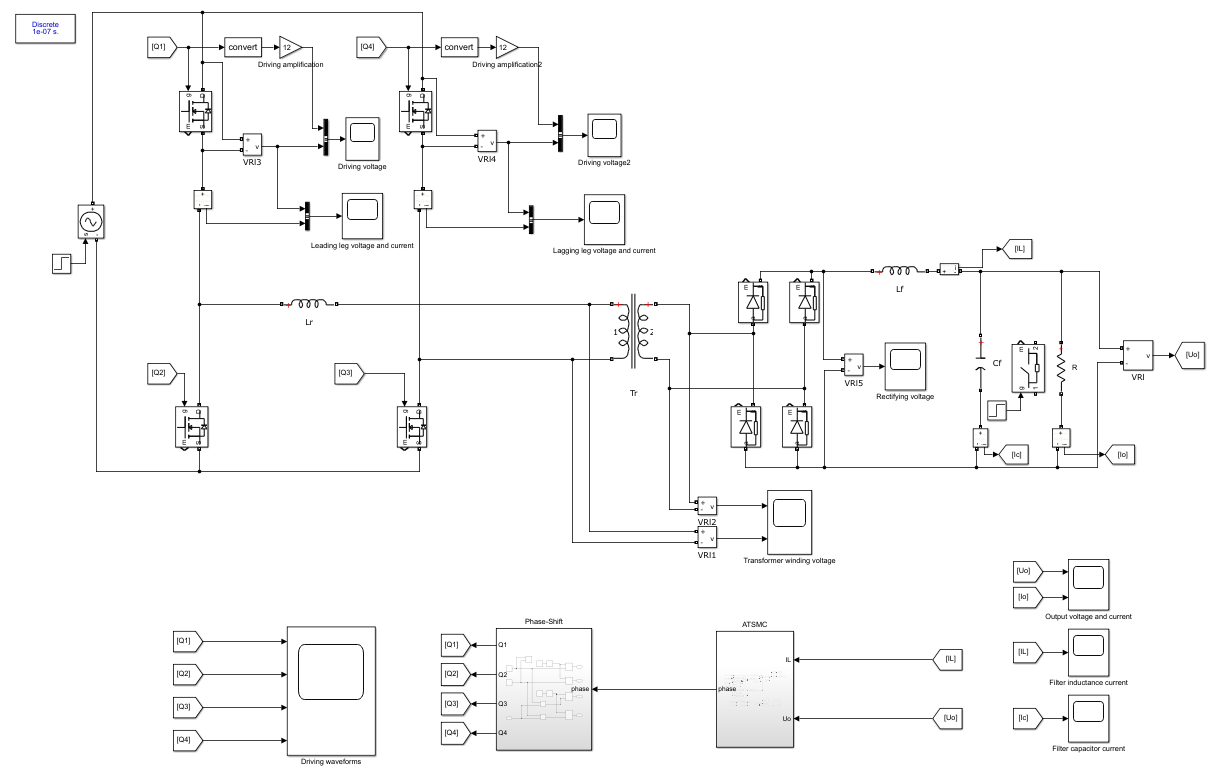

Supplement: S1 Fig — The overall design framework is given in this figure. (TIF) [file pone.0247228.s001.tif]

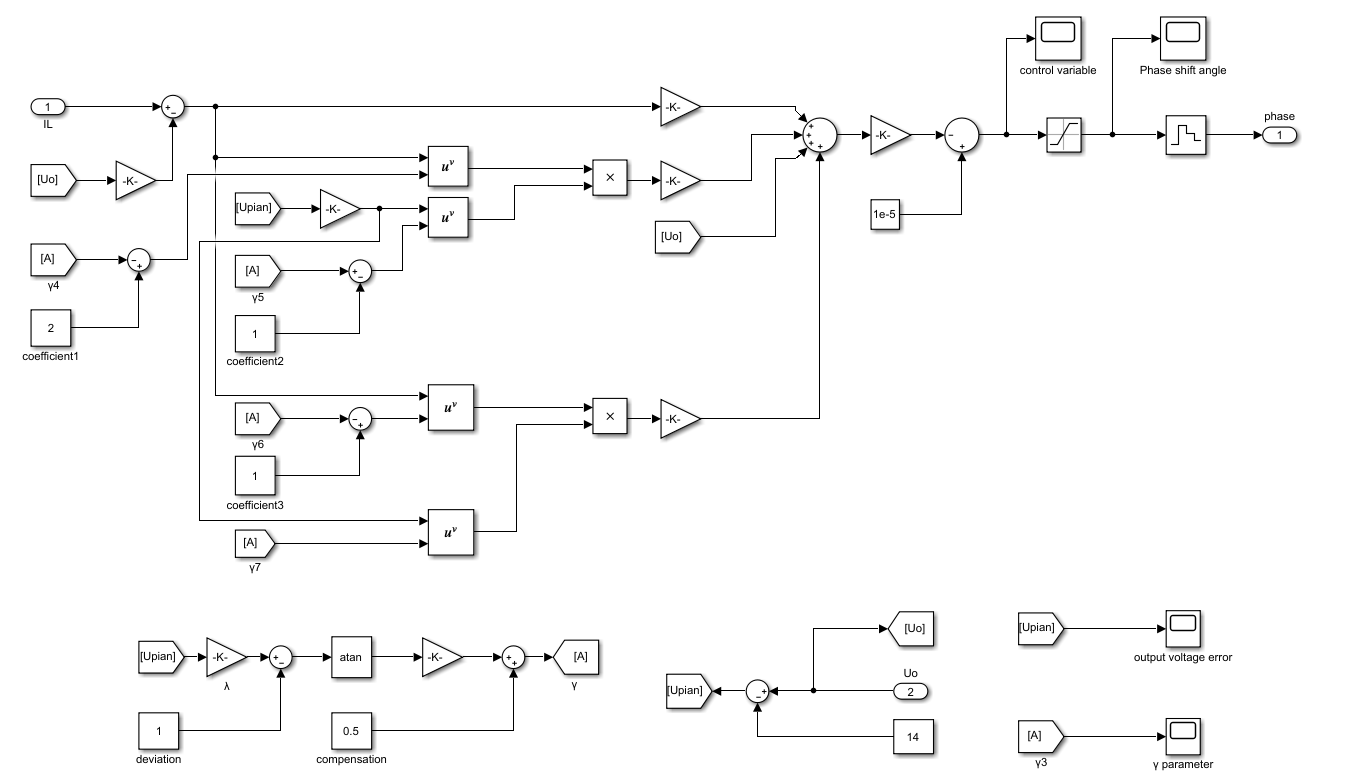

Supplement: S2 Fig — Here is the specific framework of ueq in MATLAB/Simulink. (TIF) [file pone.0247228.s002.tif]
